# Annual Acknowledgement of Reviewers

**DOI:** 10.1186/1752-0509-8-11

**Published:** 2014-02-06

**Authors:** Timothy R Sands

**Affiliations:** 1 BioMed Central, Floor 6, 236 Gray's Inn Road, London WC1X 8HB, UK

## Abstract

**Contributing reviewers:**

The editors of BMC Systems Biology would like to thank all our reviewers who have contributed to the journal in Volume 7 (2013).

## 

Amir Abdollahi

Germany

Ameeta Agarwal

USA

Balbino Alarcon

Spain

Mark Alber

USA

Réka Albert

USA

Leonidas Alexopoulos

Greece

Uri Alon

Israel

Russ Altman

USA

Grigorios Amoutzias

Greece

Gary An

USA

John Anderson

USA

Steve Andrews

USA

Kazuharu Arakawa

Japan

Masanori Arita

Japan

Luc Baeyens

Belgium

Michael Bales

USA

Julio Banga

Spain

Chris Barnes

UK

Fredrik Barrenas

USA

Eric Batchelor

USA

Daniel Beard

USA

Vincenzo Belcastro

Italy

Francois Berthiaume

USA

Katja Bettenbrock

Germany

Anastassios Bezerianos

Greece

Gyan Bhanot

USA

Sharad Bhartiya

India

Ofer Biham

Israel

Michael Blinov

USA

Joke Blom

Netherlands

Eric Blomme

USA

Sergio Bordel

Sweden

Steven Brown

USA

Sascha Bulik

Germany

Hauke Busch

Germany

Laurence Calzone

France

Yang Cao

USA

Pablo Carbonell

France

Carsten Carlberg

Finland

Javier Carrera

Spain

Michele Caselle

Italy

Ron Caspi

USA

Michele Ceccarelli

Italy

Gunnar Cedersund

Sweden

Shweta Chavan

USA

Bor-Sen Chen

Taiwan

Chun Chen

USA

Linyi Chen

Taiwan

Pao-Yang Chen

USA

William Chen

USA

Xing Chen

China

Chao Cheng

USA

Jianlin Cheng

USA

Tammy Cheng

UK

Michael Cho

USA

Murim Choi

South Korea

Hon Nian Chua

USA

Pawel Ciborowski

USA

Vily Cimpoiasu

Romania

Alys Clark

New Zealand

Daniel Coca

UK

Manuel Comabella

Spain

Greg Cooney

Australia

Carlo Cosentino

Italy

Gheorghe Craciun

USA

Travis Craddock

Canada

Peter Csermely

Hungary

Qinghua Cui

China

Zhiming Dai

China

Marc Dalod

France

Thomas Dandekar

Germany

Ramana Davuluri

USA

Rob de Boer

Netherlands

Luis de Figueiredo

UK

Alberto de la Fuente

Germany

Erik De Schutter

Japan

Gabriel del Rio

Mexico

Andrew Dewan

USA

Mario di Bernardo

Italy

Guohui Ding

China

Paul Dobson

UK

Edward Dougherty

USA

Francis Doyle III

USA

Andreas Dräger

USA

Joel Dudley

USA

Mary Dunlop

USA

Maxime Durot

France

Janusz Dutkowski

Poland

Stephen Edwards

USA

Frank Emmert-Streib

UK

Julia Engelmann

Germany

Francisco Fernández De Miguel

Mexico

Francois Fages

France

Robert Faryabi

USA

Karoline Faust

Belgium

Adam Feist

USA

Haidong Feng

USA

Farhan Feroz

UK

Roberto Ferrari

USA

Fulvia Ferrazzi

Germany

Marc Fink

USA

Christian Fleck

Netherlands

Ronan Fleming

Luxembourg

Stephen Fong

USA

Celia Fontanillo

Spain

Al Forrest

Japan

Leonid Fridlyand

USA

Karl Friston

UK

Martina Fröhlich

UK

Holger Fröhlich

Germany

Georg Füllen

Germany

André Fujita

Brazil

Atsushi Fukushima

Japan

Laura Furlong

Spain

Julien Gagneur

Germany

Kathleen Gallo

USA

Donald Gardiner

Australia

David Gfeller

Switzerland

Enrico Glaab

Luxembourg

Martin Golebiewski

Germany

Emanuel Gonçalves

UK

Andrew Goryachev

UK

Bertie Gottgens

UK

Eva Grafahrend-Belau

Germany

Bruno Gran

UK

Angela Grassi

Italy

Mariona Graupera

Spain

Bert Groen

Netherlands

Yuanfang Guan

USA

Marc Guell

USA

Natali Gulbahce

USA

Rudiyanto Gunawan

Switzerland

Zheng Guo

China

Ryan Gutenkunst

USA

Reinhard Guthke

Germany

Pietro Hiram Guzzi

Italy

Fiete Haack

Germany

Juergen Hahn

USA

Mark Hanigan

USA

Heather Harrington

UK

Leonard Harris

USA

Jan Hasenauer

Germany

Shin-Ichi Hayashi

Japan

Benjamin Heavner

USA

Matthias Heinemann

Netherlands

Tomas Helikar

USA

Bart Hendriks

USA

Henning Hermjakob

UK

Eleisa Heron

Ireland

Markus Herrgard

Denmark

James Hetherington

UK

Karsten Hiller

Luxembourg

Jean-Francois Hocquette

France

Charlie Hodgman

UK

Erhard Hofer

Austria

Kirsten Hogg

Canada

Christoph Hold

Switzerland

Rudolf Hoogenveen

Netherlands

Stefan Hoops

USA

Timothy Hore

UK

Chia-Lang Hsu

Taiwan

Pingzhao Hu

Canada

Tony Hu

USA

Yucheng Hu

China

Hsuan-Cheng Huang

Taiwan

Michael Hucka

USA

Marc-Thorsten Hütt

Germany

Y.S. Hung

Hong Kong

C. Anthony Hunt

USA

Dirk Husmeier

UK

Daniel Hyduke

USA

Dagmar Iber

Switzerland

Brian Ingalls

Canada

Francesco Iorio

UK

Liliana Ironi

Italy

David James

Australia

Neema Jamshidi

USA

Michael Janitz

Australia

Joachim Jankowski

Germany

Laura Jarboe

USA

Songshan Jiang

China

Iain Johnston

UK

Hsueh-Fen Juan

Taiwan

Jaap Kaandorp

Netherlands

Lars Kaderali

Germany

Christoph Kaleta

Germany

Atanas Kamburov

Germany

Peter Karp

USA

Tatiana Karpinets

USA

Michael Katze

USA

Joan Kelly

Australia

David Kelvin

Canada

Servé Kengen

Netherlands

Boris Kholodenko

Ireland

Dongsup Kim

South Korea

Kyung Kim

USA

John King

UK

Paul Kirk

UK

Roy Kishony

USA

Aristotelis Kittas

Germany

Gunnar Klau

Netherlands

David Klinke

USA

Edda Klipp

Germany

Andrzej Kloczkowski

USA

Yasuhiro Kobayashi

Japan

Alexey Kolodkin

Luxembourg

Natalia Komarova

USA

Mark Kon

USA

Ekaterina Kotelnikova

Russia

Frank Kramer

Germany

Andreas Kremling

Germany

Clemens Kreutz

Germany

Don Kulasiri

New Zealand

Vishwesh Kulkarni

USA

Charu Kumar

USA

Ursula Kummer

Germany

Hiroyuki Kurata

Japan

Yinglei Lai

USA

Mario Latendresse

USA

Neil Lawrence

UK

Matthew Lazzara

USA

Nicolas Le Novère

UK

Dong-Yup Lee

Singapore

Ney Lemke

Brazil

Joshua Lerman

USA

Herbert Levine

USA

Cheng Li

USA

Hongzhi Li

USA

Jiuyong Li

Australia

Lei Li

Canada

Peng Li

USA

Xia Li

China

Zheng Li

China

Haihua Liang

USA

James Liao

USA

Wolfram Liebermeister

Germany

Bing Liu

Australia

Fen Liu

China

Guimei Liu

Singapore

Juan Liu

China

Wei Liu

USA

Wei-Chung Liu

Taiwan

Zhi-Ping Liu

China

Daniel Machado

Portugal

Michael Mackey

Canada

Rodrigo Maillard

USA

Ryszard Maleszka

Australia

Michael Mangold

Germany

Long Mao

China

Xizeng Mao

USA

Costas Maranas

USA

Daniel Marbach

USA

Anna Marciniak-Czochra

Germany

Beurton-Aimar Marie

France

Olivier Martin

France

Ali Masoudi-Nejad

Iran

Yukiko Matsuoka

Japan

Randolph Matthews

USA

Jean-Pierre Mazat

France

Orianne Mazemondet

Germany

Ramit Mehr

Israel

Pankaj Mehta

USA

Pedro Mendes

UK

Anand Merchant

USA

Mutlu Mete

USA

Aline Metris

UK

Tijana Milenkovic

USA

Brooke Miller

USA

Ron Milo

Israel

Gary Mirams

UK

Markus Mohaupt

Switzerland

Jaap Molenaar

Netherlands

Steffen Möller

Germany

John Morgan

USA

Sandro Morganella

Italy

Andrew Mouland

Canada

Ralf Mrowka

Germany

Sach Mukherjee

UK

T.M. Murali

USA

David Murrugarra

USA

Syed Murtuza Baker

Germany

Chris Myers

USA

Ganesh Nagaraju

India

Seungyoon Nam

South Korea

Manikandan Narayanan

USA

Ali Navid

USA

Satyaprakash Nayak

USA

David Nickerson

New Zealand

Zoran Nikoloski

Germany

Nasimul Noman

Japan

Intawat Nookaew

Sweden

Raimund Ober

USA

Eitan Okun

Israel

Michael Oldham

USA

Guilherme Oliveira

Brazil

Brett Olivier

Netherlands

Shuichi Onami

Japan

Yury Orlov

Russia

Juan Ortin

Spain

Richard Orton

UK

Irene Otero Muras

Spain

Diego Oyarzun

UK

Andrea Pagnani

Italy

Jürgen Pahle

UK

Tun-Wen Pai

Taiwan

Bernhard Palsson

USA

Yi Pan

USA

Casian Pantea

USA

Irene Papatheodorou

UK

Sunghyouk Park

South Korea

Joel Parker

USA

John Parkinson

Canada

Kiran Raosaheb Patil

Germany

Josch Pauling

Denmark

Georgios Pavlopoulos

Greece

Andy Perkins

USA

Edward Perkins

USA

Theodore Perkins

Canada

Andrew Phillips

USA

Esa Pitkänen

Finland

Christopher Previti

Norway

Nathan Price

USA

Jeff Prideaux

USA

Carole Proctor

UK

Teresa Przytycka

USA

Xiaoning Qian

USA

Lake-Ee Quek

Australia

Francisco Quintana

USA

Ovidiu Radulescu

France

Andreas Raue

Germany

Gabe Redding

New Zealand

Jennifer Reed

USA

Erzsébet Regan

USA

Marc Rehmsmeier

Norway

Gene Robinson

USA

Isabel Rocha

Portugal

Miguel Rocha

Portugal

Maria Rodriguez-Fernandez

USA

Jim Rogers

USA

Philip Rosenthal

USA

Sergio Rossell

Netherlands

Jianhua Ruan

USA

Cristina Rubio-Escudero

Spain

Ann Rundell

USA

Peter Ruoff

Norway

Ravi Sachidanandam

USA

Julio Saez-Rodriguez

UK

Shigeru Saito

Japan

Howard Salis

USA

Tapesh Santra

Ireland

Vinay Satish

USA

Jeffrey Saucerman

USA

Herbert Sauro

USA

Arnold Saxton

USA

Jörg Schaber

Germany

Maria Schilstra

UK

Joep Schmitz

Netherlands

Konstantin Schneider

Germany

Markus Scholz

Germany

Dietmar Schomburg

Germany

Falk Schreiber

Germany

Klaus Schughart

Germany

Marvin Schulz

Germany

Nicola Segata

Italy

Lorenzo Sempere

USA

Rodolphe Sepulchre

Belgium

Jun Sese

Japan

Vahid Shahrezaei

UK

Ekaterina Shelest

Germany

Bairong Shen

China

Hiroshi Shimizu

Japan

Benjamin Shoemaker

USA

Heike Siebert

Germany

Alina Sîrbu

Ireland

Jan Skotheim

USA

Kieran Smallbone

UK

Lucian Smith

USA

Harry Sokol

France

Alexey Solovyev

USA

Joe Song

USA

Hermona Soreq

Israel

Ulrich Stelzl

Germany

Brandilyn Stigler

USA

Ronny Straube

Germany

Takeyuki Sugiura

Japan

Zhirong Sun

China

Zhongyao Sun

USA

William Swindell

USA

Leila Taher

Germany

Kazuhiro Takemoto

Japan

Gouhei Tanaka

Japan

Chao Tang

USA

Christof Taxis

Germany

Ronald Taylor

USA

Marinus te Pas

Netherlands

Andrew Teschendorff

UK

Fabian Theis

Germany

Günter Theißen

Germany

Nathalie Theret

France

Johan Thevelein

Belgium

Denis Thieffry

France

Ines Thiele

Iceland

Weidong Tian

China

Chabane Tibiche

Canada

Takashi Tokino

Japan

Cong Trinh

USA

Shahab Uddin

Saudi Arabia

Jitka Ulrichová

Czech Republic

Korkut Uygun

USA

Natal van Riel

Netherlands

Matthieu Vignes

France

Alejandro Fernández Villaverde

Spain

Ganesh Viswanathan

India

Eberhard Voit

USA

Steffen Waldherr

Germany

Dirk Walther

Germany

Edwin Wang

Canada

Feng-Sheng Wang

Taiwan

Guanyu Wang

USA

Jin Wang

USA

Junwen Wang

China

Peng Wang

China

Xiyin Wang

USA

Ya Wang

USA

Yong Wang

China

Patrick Warren

UK

Mark Weatherall

New Zealand

Wilfried Weber

Germany

Erik Welf

USA

Thomas Werner

Germany

Thomas Wilhelm

UK

John Williams

USA

Wynand Winterbach

Netherlands

Aaron Wise

USA

Ulrike Wittig

Germany

Olaf Wolkenhauer

Germany

Clemens Wrzodek

Germany

Fang-Xiang Wu

Canada

Hulin Wu

USA

Ming Wu

USA

Wei-Sheng Wu

Taiwan

Stefan Wuchty

USA

Ioannis Xenarios

Switzerland

Jingfa Xiao

China

Zhi Xie

USA

Dong Xu

USA

Yoshihiro Yamanishi

Japan

Guiying Yan

China

Jinn-Moon Yang

Taiwan

Ling Yang

China

Zhanjiang Yuan

China

Hong Yue

UK

Chiou-Hwa Yuh

Taiwan

Naif Zaman

Canada

Hossein Zare

USA

Alex Zelikovsky

USA

Tao Zeng

China

Bing Zhang

USA

Fuzhong Zhang

USA

Michael Zhang

USA

Shihua Zhang

China

Wanwei Zhang

China

Zhaolei Zhang

Canada

Xingming Zhao

China

Fengfeng Zhou

China

Qianqian Zhu

USA

Zhihao Zhuang

USA

Zhike Zi

Germany

Andrei Zinovyev

France

Ali Zomorrodi

USA

Jinfeng Zou

China

Xiufen Zou

China

